# Decision-making for indoor residual spraying in the post-elimination phase of visceral leishmaniasis in Nepal

**DOI:** 10.1371/journal.pntd.0014355

**Published:** 2026-05-18

**Authors:** Megha Raj Banjara, Murari Lal Das, Siddharth Joshi, Krishna Raj Pant, Uttam Raj Pyakurel, Gokarna Dahal, Axel Kroeger, Abraham Aseffa, Christine M. Halleux, Anand Ballabh Joshi

**Affiliations:** 1 Central Department of Microbiology, Tribhuvan University, Kirtipur, Kathmandu, Nepal; 2 UNICEF/UNDP/World Bank/WHO Special Programme for Research and Training in Tropical Diseases, World Health Organization, Geneva, Switzerland; 3 Member, Regional Technical Advisory Group (RTAG) for Kala-azar and Malaria, WHO-SEARO, New Delhi, India; 4 Public Health and Infectious Disease Research Center (PHIDReC), Kathmandu, Nepal; 5 Epidemiology and Disease Control Division, Department of Health Services, Teku, Kathmandu, Nepal; 6 Centre for Planetary Health, Albert-Ludwigs-University, Freiburg, Germany; Pure Earth, UNITED STATES OF AMERICA

## Abstract

**Background:**

The effectiveness of indoor residual spraying (IRS) during the post elimination phase of visceral leishmaniasis (VL) and when to stop its application is uncertain. This study investigated the relationship between VL occurrence and frequency of IRS on vector density, infection rates, and insecticide susceptibility.

**Methodology/principal findings:**

Four villages in the Sarlahi district served as sentinel surveillance sites for sandfly density measurement and xeno-monitoring, selected based on VL endemicity levels of high, moderate, low and non-endemic (no reported cases in the past 10 years). A random sub-sample of households from each village was selected for sandfly surveillance. The sample size of 380 was determined to detect a 1% infection rate of sandflies with 95% confidence interval. Ecological and epidemiological data were collected and IRS activity data between 2012–2023 was analysed. Sandflies were collected using CDC light traps and mouth aspirators for 12 months from March 2023 to February 2024 and tested with PCR for kDNA of *Leishmania donovani*. Monthly density of female *Phlebotomus argentipes* sandflies varied across endemicity levels, with a peak in November, with fluctuations observed throughout the year. Village wise sandfly pools positivity with parasite DNA varied with level of endemicity (66.7% in high VL endemicity villages, 62.1% in moderate, 36.8% in low, and 26.3% in non-endemic villages). Overall, among the total 91 pools of sandflies tested, 50.5% were positive for parasite DNA. *P. argentipes* showed high susceptibility to insecticides alpha-cypermethrin, bendiocarb, deltamethrin, and malathion. There were differences in IRS applications with variations in coverage and frequency and programmatic factors across municipalities, with no IRS conducted in some villages. Occupational distribution varied across endemicity levels, and there were differences in sleeping habits during warm weather. Non-impregnated bed nets were available across all endemicity levels.

**Conclusions/significance:**

IRS should be continued in the villages based on surveillance of sandfly density and reports of VL cases in the post elimination phase of VL.

## Author summary

Indoor residual spraying (IRS) remains a main vector control strategy for visceral leishmaniasis (VL) elimination. The VL cases have decreased significantly in Nepal; however, when to stop IRS is an important question. This study was conducted to analyze entomological factors, occurrence of VL and, programmatic factors to recommend the application of IRS. The sandfly density was found to be high in VL endemic and non-endemic areas and likewise the parasite positivity determined by xenomonitoring was high. *Phlebotomus argentipes* were susceptible to a number of insecticides tested. The application of IRS by the national programme was found to be limited. The communities had risk behaviours and practices for VL occurrence. Therefore, IRS should be continued in the villages with reports of VL cases and surveillance of vector sandfly.

## Introduction

Annually, an estimated 50,000–90,000 new cases of visceral leishmaniasis (VL, also known as kala-azar) emerge globally, yet only 25–45% are reported to the World Health Organization (WHO) [1]. In the context of South-East Asia, the WHO is supporting the initiative to eliminate VL during 2022–2026. The goal is to reduce incidence to less than 1 case per 10,000 inhabitants at the sub-district level in Bangladesh and India, and at district level in Nepal [1].

Nepal has updated the endemicity status of its districts, identifying 47 out of 77 districts as endemic for VL. In 2023, two districts (implementation units in the country) recorded rates above the elimination threshold of 1 per 10,000 population [1]. Although the number of VL cases has declined significantly, the last phase of elimination is often difficult. Since 2013, Nepal has reduced the VL incidence to the target threshold [2]; however, VL emerged in new districts posing challenges to the verification of elimination. These challenges include epidemiological shifts in VL [3,4], the geographical expansion of the disease [5–7], relapses [8], climate change [9], and other issues such as limited community awareness, poor implementation of vector control interventions, and inadequate diagnostic and treatment services in newly VL reporting districts.

The proven VL vector in Nepal and neighbouring countries is *Phlebotomus argentipes* [10,11].Vector control and treatment of VL patients are the main strategies for VL elimination in the Indian subcontinent. The national program of Nepal and other countries of Southeast Asia have focused on vector control through indoor residual spraying (IRS) and the distribution of long-lasting insecticidal nets (LLINs). Varying effectiveness of IRS have been reported in the controlled experiments and real life situations [12–14]. Although research has been conducted on impregnation of existing bed nets with insecticide KOTAB 123, durable wall linings in households, insecticidal wall painting with Inesfly paint, and other environmental measures [15–18], these tools are not used in practice yet for vector control. IRS is a crucial tool in vector control for the elimination of VL, primarily due to the indoor resting habits of sandflies. Pyrethroids are being used for IRS across the Indian subcontinent. Currently, IRS is recommended twice a year in villages that have reported at least one case of VL in the past three years. The effectiveness of IRS in vector control and reduction of VL cases is an ongoing discussion [19]. Studies in India and Nepal demonstrated that IRS has reduced substantial sandfly abundance contributing to reductions in VL cases [20–22]. One study also demonstrated no associations between humidity, rainfall, and temperature on VL incidence [22]. Previous studies in Nepal have primarily focused on the reduction of vector density as the primary outcome of interest; however, there exists a gap in understanding the relationship between vector control measures and their impact on reducing the VL burden. Although VL incidence has significantly declined, sustaining elimination in the pre-elimination phase remains a major challenge. There is limited information on when to cease IRS after VL elimination and on its overall value in eliminating VL. Moreover, there is a lack of systematic assessment of IRS, including vector and VL transmission parameters in humans, to provide recommendations for different transmission scenarios.

Some villages of Nepal, previously endemic for VL, have not reported VL cases after IRS interventions for many years [2]. The impact of IRS can be reverted after stopping IRS as VL resurged after 10–15 years in India [22]. Therefore, comparing the status of VL and vector density between districts with or without VL, including their history of IRS, could provide valuable insights into the role of IRS. The preventive measures, such as IRS, applied over many years in these villages would help analyze factors contributing to VL occurrence in specific districts.

The diversity and density of VL vectors has been studied in Nepal since 1980. Measuring vector density in areas with varying levels of VL endemicity would help in determining the threshold of vector density that indicates potential risk of outbreaks or identifies outbreak-prone areas. In non-endemic areas, vector control activities might not be necessary. This vector density data could provide critical information to help decide when to stop IRS in particular areas. Xenomonitoring of sandflies collected from areas with different endemicity status can provide information on infection rates in sandflies, thereby elucidating their role in VL transmission. The success of an IRS program mostly depends on the vector’s response to specific insecticides and their resting behavior. Insecticide susceptibility tests of VL vectors have been performed frequently in India [23], Bangladesh and Nepal [24] to take necessary actions in time if resistance is reported. All these tests were performed according to WHO guidelines developed for susceptibility testing of mosquitoes [25,26]. Though instruction for susceptibility testing of sandfly was provided [27], discriminating concentrations indicated for mosquitoes were used for sandflies before 2023. In 2022, Nepal reported that *P. argentipes* sandflies from both IRS treated and untreated villages showed resistance to alpha-cypermethrin 0.05% and suggested to switch to alternative classes of insecticides for IRS [28]. But in 2023 discriminating concentration of alpha-cypermethrin for *P. argentipes* was changed to 0.1% by WHO [29]. Hence, an update on this status is required using the new WHO guideline to guide vector control services in adjusting the insecticides in use. The overall objective of this study was to identify critical determinants that can inform rational decision-making on the application of IRS in the pre- and post-elimination phases of visceral leishmaniasis.

## Materials and methods

### Ethical consideration

This study received ethical approval from Nepal Health Research Council (Regd. No.: 363/2022 P). Written informed consent was obtained from the household heads for vector survey activities. For collection of data on households’ characteristics and people’s behaviour on VL preventive measures, written consent was obtained before the interview of the participants. Anonymity of data was maintained for confidentiality of information. Child participants were not included in the study.

### Study design

**Fig 1:**
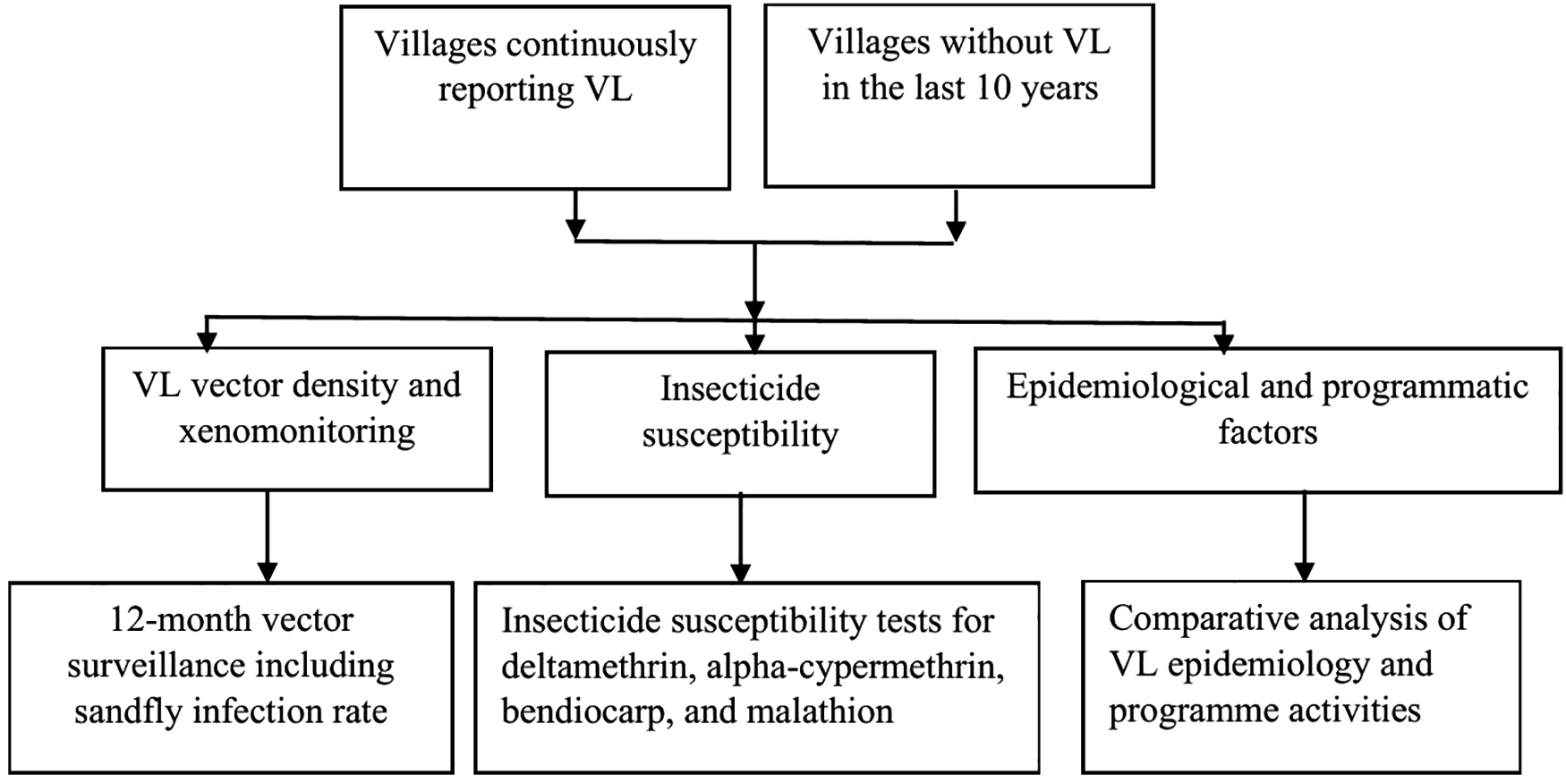
Diagrammatic representation of the study design.

This implementation research was conducted to investigate the impact of indoor residual spraying on the occurrence of VL along with VL vector abundance, infection rate in sandflies, and insecticide susceptibility. Information on IRS activity from 2012 to 2023 was obtained from Sarlahi Health Office. Vector density and sandfly infection rates were monitored over one year (2023–2024), and insecticide susceptibility was also studied during the same period (Fig 1).

Data on ecological factors, including temperature, rainfall, humidity, and the presence of pastures, forests, and urban proximity were collected. Epidemiological data (both historical and current) and programmatic factors related to VL control or elimination were assessed in four villages in Sarlahi district with varying levels of endemicity.

### Study sites and population

Four villages in Sarlahi district were selected as sentinel surveillance sites for sandfly density measurement and xenomonitoring, based on their VL endemicity: i) high VL endemic, ii) moderate VL endemic, iii) low VL endemic, and iv) non-endemic (no VL cases during the past 10 years). VL endemicity was determined using national surveillance data from the past decade, categorizing villages as high (>66th percentile), moderate (33–66th percentile), and low (<33rd percentile). All households of the village were covered in the study whereas a sub-sample of households was selected from each village for entomological surveillance.

The selected villages and their characteristics were: Ishworpur Ward No. 2 (Writerkhor), with high VL endemicity, 99 households in the village, and at an altitude of 144.7 meters; Kabilasi ward No. 10 (Salimpur), with moderate VL endemicity, 133 households in the village, at an altitude of 112.6 meters; Bagmati ward No. 9 (Shankarpur), with low VL endemicity, 171 households in the village, at 126.1 meters altitude; and Kadauna ward No. 4 (Motipur), a non-endemic village for VL, with 251 households, located at 100 meters altitude.

### Sample size and sampling

A previous study from Nepal reported less than 1% infection rates in sandflies [10]. We expected a 1% (0.01) infection rate of sandflies in endemic villages and aimed to detect this with a 95% confidence level and a 2% (0.02) margin of error. The sample size was calculated using the formula n = Z^2^.p.(1-p)/ E^2^, where α = 0.025, Z = 1.96, p = 0.01, and E = 0.02. Based on this calculation, a total of 380 sandflies were collected across four villages, with 95 sandflies sampled from each village.

Additionally, the expected positive results (infected sandflies) in the sample were 4. We assumed an average capture rate of 5 sandflies per night per trap, this equates to 12 sandflies from the 1 trap per household (HH) and 48 sandflies from the 12 study HHs per village. Including 3 endemic villages and 1 non-endemic village, we anticipated capturing 240 sandflies per month totaling 2880 sandflies per year, with an expectation of 29 infected sandflies.

All households of each village were invited for a survey for data collection on their household characteristics and people’s behaviour. For entomological investigations, twelve households (one VL index case household and 11 households around it and within 50m radius and 12 randomly selected households for VL non-endemic village) were selected for vector surveillance and xenomonitoring.

### Epidemiological data collection

VL case data from the past ten years were obtained from the Sarlahi District Health Office and the Epidemiology and Disease Control Division, Ministry of Health, ensuring confidentiality. The research assistant, under the PI’s supervision, identified households with reported cases and verified each case individually. The household head (who, in the Nepalese society, is the primary decision-maker in the household) was determined by asking family members. The research assistant informed the household head about the study’s objectives, procedures, and benefits, and requested their participation. Individual case-level information was not collected. Demographic, socio-economic, geographic, and other characteristics of households with reported VL cases in these villages were collected through interviews with the household head after obtaining written informed consent. Similar information, including family size, type of house, and the surrounding environment, was also collected from households in villages without current VL cases.

### Ecological data collection

During the 12-month vector surveillance, data on temperature, rainfall, and humidity were recorded at the sentinel sites on the days of collection of sandflies (two days per village per month), along with information on household characteristics. Temperature and humidity were recorded using thermohygrometer (HTC-1, Shenzhen Bestone Industrial Co. Ltd. Shenzhen, China) and rainfall was recorded using a rain gauge.

### Identification of sandfly and measurement of density

A trained entomological survey team conducted 12 months of vector surveillance using CDC light traps (John W. Hock Co., Gainesville, FL) to measure vector density over two consecutive nights in the study area [30]. In each of the selected house, one light trap was placed inside the bed room of the selected houses, on a fixed place every month. Trained personnel, under the guidance of an entomologist, collected sandfly vectors as part of the study. Light traps were placed in a corner of the house from 6 PM to 6 AM, positioned 2 inches from the wall and 6 inches above the floor. Additionally, sandflies were collected from selected households with cattle sheds, kennels, and poultry houses using manual aspirators. Two insect collectors manually collected sandflies for two hours in the morning from 6 to 8 AM using manual aspirators in the same households and premises.

Bags from the traps were placed in a refrigerated container and transported to the field station, where sandflies were triaged, identified, and separated under a stereoscopic microscope. Sandfly density was calculated as the mean number of sandflies per CDC light trap per household per night and per man-hour per village. Morphological characters were used for identification of species of sandflies in the field using different keys [31–33].

### Xenomonitoring of sandflies

Collected sandflies were preserved in 70% alcohol and transported to the Decode Genomics and Research Center laboratory. Village-wise pools of 5 *P. argentipes* were prepared. Parasite DNA was extracted using the QIAamp DNA Mini Kit (Qiagen, Germany, Lot No: 51304). The kDNA of *L. donovani* was amplified with species-specific PCR (Macrogen, Korea) using primers targeting kDNA (LIN Forward: 5’-GGG GTT GGT GTA AAA TAG GG-3’; LIN Reverse: 5’-CAG AAC GCC CCT ACC CG-3’), which amplify a 720 bp PCR product. The temperature profile for the reaction included initial denaturation at 95^o^C for 10 min (1 cycle), and denaturation at 94^o^C for 30 seconds, annealing at 63^o^C for 90 seconds, extension at 72^o^C for 90 seconds for 40 cycles and final extension at 72^o^C for 10 minutes [34].

Each PCR reaction was performed in a total volume of 25 µl, consisting of 6 µl master mix (Solis BioDyne, Estonia, Cat No: 04-25-00115), 1 µl each of forward and reverse primers, 4 µl DNA template, and 13 µl distilled water. The PCR product was analyzed using a 1% agarose gel.

### Assessment of insecticide susceptibility

Field-collected adult female *P. argentipes* were used for this study. Pyrethroids (0.10% alpha-cypermethrin, 0.25% and 0.05% deltamethrin), carbamate (0.1% bendiocarb), and organophosphate (5% malathion) were tested to assess sandfly susceptibility. The insecticide resistance test was performed according to the WHO tube test method [29]. Sandflies were collected using manual aspirators from inside human dwellings and cattle sheds in unsprayed villages during the day. Data were recorded using a specific form.

*Bioassay procedure:* The WHO bioassay test kit, insecticide impregnated papers, silicon oil papers (for pyrethroids), olive oil papers (for carbamates and organophosphates) and control papers were procured from Universiti Sains Malaysia, Penang, Malaysia (WHO recommended organization) to conduct standardized susceptibility tests on sandflies.

Silicone oil-impregnated papers were used as controls for pyrethroid bioassays, while olive oil-impregnated papers were used as controls for organophosphate and carbamate bioassays to ensure that any observed effects on sandflies were attributable to the insecticides being tested.

*P. argentipes* were collected from the animal sheds in a chamber using mouth aspirators. The chamber was kept for 2 hours in the field laboratory for starvation. After that time 20–25 of them were transferred to each holding tubes with control papers of respective insecticide and kept for 60 minutes holding period. They were then transferred from the holding tubes to four exposure tubes lined with insecticide impregnated papers. Two control tubes with plain papers were used with each set of test. The exposure period was 60 minutes. Knockdown of the sand flies were recorded at 10, 15, 20, 30, 40, 50, and 60 minutes after the start of the exposure. After 60 minutes of exposure, the sand flies were transferred back to the holding tubes, provided with 10% glucose-soaked cotton wool pads on the fine mesh screen, and placed in an area with diffused illumination for the next 24 hour recovery period. To stabilize the ambient temperature below 30^o^C and the relative humidity around 80%, the holding tubes were placed in a container box covered with wet towels. The numbers of dead and alive sand flies from each of the exposure and control holding tubes were counted and recorded at the end of the recovery period. After a 24 hour recovery period, the test was considered valid; if control mortality was < 5%; mortality was corrected by Abbotts’ formula [35] if control mortality was > 5% and < 20%; and the test was repeated if control mortality was > 20%.

### Assessment of programmatic factors

Data on VL patients reported from endemic areas and IRS information from 2012 to 2022 were obtained from the Epidemiology and Disease Control Division, Ministry of Health. Researchers from PHIDReC, Nepal, conducted a retrospective analysis of epidemiological, entomological, and ecological survey data, along with VL case data from the past decade. Programmatic data included information on the application of IRS, other vector control interventions, active case detection, and housing projects implemented by the government or other agencies. This data was collected through interviews with the vector control officer of the District Health Office, the focal person for vector-borne diseases. The interviews, conducted in the officer’s workspace, lasted approximately 30 minutes, during which the officer reviewed records to provide details on vector control and VL case detection activities.

### Data management and analysis

A well-validated data entry program was developed using Epi Info (Version 7), with all data double-entered to ensure accuracy. Data quality was maintained according to established management guidelines. SPSS version 25 was used for the analysis of data. Descriptive statistics frequencies and percentages were calculated for description of demographic characteristics, risk factors of VL, awareness of VL, and insecticide susceptibility of village per arm in different endemicity. Vector density, temperature, rainfall, and humidity were presented in mean with standard deviation. Association of variables with proportions were calculated using Chi-square test. p-values less than 0.05 were considered statistically significant. All comparisons were presented based on level of VL endemicity of the villages.

For the qualitative analysis, a thematic approach was used to analyze data. Prior to data collection, all team members were briefed on the study objectives, consent process, interview guidelines, and the importance of data quality. The principal investigator and co-investigator observed interviews, conducted peer debriefing, checked collected data, performed transcription and translation, and carried out triangulation and member checking to identify inconsistencies. Data was organized using Atlas.

## Results

### Characteristics of the population in the study sites

Among the four villages selected for the study, majority of the residents were unskilled workers, housewives and students. In VL high and moderate endemic villages, 54.5% and 55.6% people respectively slept on the floor and only 49.5% and 56.4% people had used bednets the previous night. However, 100% people of VL low endemic and 85.7% of non-endemic villages slept on the bed and 100% people of VL low endemic village and 91.2% of non-endemic village slept under the bednets last night (S1 Table).

### Analysis of risk factors for VL

Number of rooms in the households were categorized into three groups: one to two rooms, three to five rooms, and six or more rooms. The percentage of households in each category varied across endemicity levels. Non-endemic villages had a higher percentage of households (60.9%) with one to two rooms compared to endemic villages (41.8%). Similarly, the distribution of households with three to five rooms and six or more rooms differed across villages with varying endemicity. The median number of rooms per household also varied (3 rooms in endemic villages and 2 rooms in non-endemic villages), reflecting differences across endemicity levels (S2 Table).

In households with verandas, residents often sleep outside during the warm season. We inquired whether households had a veranda and if residents used it for sleeping during warm weather. The distribution of these characteristics varied significantly across endemicity levels, with notable differences in sleeping arrangements during the warm season. Statistical significance of these differences was determined using p-values, with p < 0.05 indicating significant differences between kala-azar endemic and non-endemic villages (Tables 1 and S2).

**Table 1:** Bednet usage among households in VL endemic and non-endemic villages.

Characteristics (*n*, %)	Ishworpur Ward No. 2 (Writerkhor) (High endemic)	Kabilasi Ward No. 10 (Salimpur) (Moderate endemic)	Bagmati Ward No. 9 (Shankarpur) (Low endemic)	Kadauna Ward No. 4 (Motipur) (Non-endemic)	Endemic (n = 98)	Non-endemic (n = 23)	p-value
*HHs with verandas and sleeping in warm season*							
Yes	5 (29.4)	6 (11.1)	3 (11.1)	11 (47.8)	14 (14.3)	11 (47.8)	0.001
No	12 (70.6)	48 (88.9)	24 (88.9)	12 (52.2)	84 (85.7)	12 (52.2)	
*Bednet in the house*							
Yes	14 (82.4)	51 (94.4)	27 (100.0)	20 (87.0)	92 (93.9)	20 (87.0)	0.254
No	3 (17.6)	3 (5.6)	0 (0.0)	3 (13.0)	6 (6.1)	3 (13.0)	
*No. of bednets in the house*							
No bednet	3 (17.6)	3 (5.6)	0 (0.0)	3 (13.0)	6 (6.1)	3 (13.0)	0.467
1 bednet	8 (47.1)	20 (37.0)	0 (0.0)	8 (34.8)	28 (28.6)	8 (34.8)	
2 bednets	5 (29.4)	24 (44.4)	7 (25.9)	7 (30.4)	36 (36.7)	7 (30.4)	
3 bednets	0 (0.0)	4 (7.4)	9 (33.3)	4 (17.4)	13 (13.3)	4 (17.4)	
≥ 4 bednets	1 (5.9)	3 (5.6)	11 (40.7)	1 (4.3)	15 (15.3)	1 (4.3)	
*Seasonal use of bednets*							
Summer	14 (82.3)	16 (29.6)	15 (55.6)	12 (52.2)	45 (45.9)	12 (52.2)	0.419
Winter	0 (0.0)	0 (0.0)	1 (3.7)	1 (4.3)	1 (1.0)	1 (4.3)	
Always	3 (17.6)	38 (70.4)	11 (40.7)	10 (43.5)	52 (53.1)	10 (43.5)	

Bednet (both commercial ordinary bednets and long-lasting insecticidal bednets) use in both VL endemic and non-endemic villages was found to be above 85%. 13% of households in VL non-endemic villages had no bednets whereas 6.1% households in VL endemic villages had no bednets in their house. Likewise, 99% of people of VL endemic villages and 95.7% of people of VL non-endemic village used bednets in summer whereas 53.1% of people of endemic villages and 43.5% of people of non-endemic villages always used bednets (Table 1).

The awareness of kala-azar among household heads of endemic villages was only 45.9% and it was only 13% in non-endemic villages. The majority of people (79.6% from endemic and 82.6% from non-endemic villages) did not know the symptoms of kala-azar. Similarly, most of the people did not know the transmission routes of kala-azar (Table 2).

**Table 2:** Awareness of VL (kala-azar) among household heads.

Characteristics	Ishworpur Ward No. 2 (Writerkhor) (High endemic)	Kabilasi Ward No. 10 (Salimpur) (Moderate endemic)	Bagmati Ward No. 9 (Shankarpur) (Low endemic)	Kadauna Ward No. 4 (Motipur) (Non-endemic)	Endemic (n = 98)	Non-endemic (n = 23)	p-value
*Heard of kala-azar*							
Yes	13 (76.5)	10 (18.5)	22 (81.5)	3 (13.0)	45 (45.9)	3 (13.0)	0.004
No	4 (23.5)	44 (81.5)	5 (18.5)	20 (87.0)	53 (54.1)	20 (87.0)	
*Knowledge of symptoms of kala-azar*							
Fever	5 (29.4)	0 (0.0)	8 (29.6)	4 (17.4)	13 (13.3)	4 (17.4)	0.1692
Enlargement of spleen	6 (35.3)	0 (0.0)	0 (0.0)	0 (0.0)	6 (6.1)	0 (0.0)	
Blackening of skin	0 (0.0)	0 (0.0)	1 (3.7)	0 (0.0)	1 (1.0)	0 (0.0)	
Do not know	6 (35.3)	54 (100)	18 (66.7)	19 (82.6)	78 (79.6)	19 (82.6)	
*Kala-azar is a curable disease*							
Yes	8 (47.1)	5 (9.3)	15 (55.6)	0 (0.0)	28 (28.6)	0 (0.0)	<0.001
No	4 (23.5)	45 (83.3)	6 (22.2)	21 (91.3)	55 (56.1)	21 (91.3)	
Do not know	5 (29.4)	4 (7.4)	6 (22.2)	2 (8.7)	15 (15.3)	2 (8.7)	
*Knowledge on transmission of kala-azar*							
Contaminated/polluted water	0 (0.0)	0 (0.0)	0 (0.0)	0 (0.0)	0 (0.0)	0 (0.0)	<0.001
Mosquito bites	5 (29.4)	0 (0.0)	13 (48.1)	1 (4.3)	18 (26.9)	1 (4.3)	
Sandfly bites	0 (0.0)	0 (0.0)	0(0.0)	0 (0.0)	0 (0.0)	0 (0.0)	
Do not know	12 (70.6)	54 (100)	14 (51.9)	22 (95.7)	16 (23.9)	22 (95.7)	

### Abundance, infection rates and insecticide susceptibility status of *P. argentipes*

**Fig 2:**
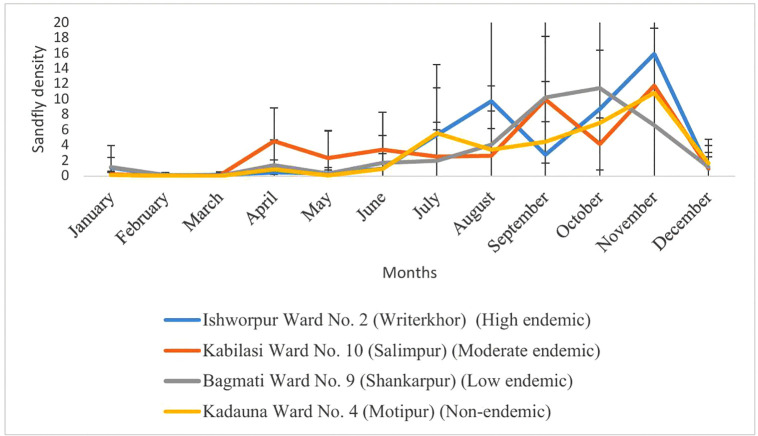
Monthly density of female *P. argentipes* sandfly collected in VL endemic and non-endemic villages (per CDC light trap per night per house).

In this study, the temperature during the study period ranged from 15-36^o^C and humidity ranged from 23-99% (S3 Table). The seasonal and monthly variations in the density of the female *P. argentipes* vector varied across different endemicity status, generally high density in November and low in February and March. There was an increase in *P. argentipes* density from July. High endemic village (Writerkhor) showed low to moderate density of female *P. argentipes* with fluctuation across the months. Moderate endemic village (Salimpur) showed variable densities across the months, with peak observed in certain months. Low endemic village (Shankarpur) showed moderate densities, with fluctuation throughout the year. Non endemic village (Motipur) generally had low densities, with some months reporting no or minimal vector collection (Fig 2)).

The field collected *P. argentipes* from other villages were used for insecticide susceptibility tests. The overall knock down of *P. argentipes* during a one hour exposure period was 90% (95% CI = 84.1-95.8) for deltamethrin (0.05%), 100% for deltamethrin (0.25%), 91% (95% CI = 85.3-96.6) for alphacypermethrin (0.10%), 100% for bendiocarb (0.10%), and 91% (95% CI = 85.3-96.6) for malathion (5.0%). The mortality of *P. argentipes* after a 24 hour recovery period was 100% for all the insecticides tested (Table 3).

**Table 3:** Insecticide susceptibility of *P. argentipes* sandflies.

Insecticides	Concentration (%)	Month of testing	Mean temperature (°C)	Mean humidity (%)	No. of sandflies exposed	Knock down during one hour exposure period (%)	Mortality after 24 hours recovery period (%)
*Pyrethroids*			Min. = 28.54 Max. = 29.30	Min. = 79.0 Max. = 85.8			
Deltamethrin	0.05	August			100	90	100
Deltamethrin	0.25	August			100	100	100
Alphacypermethrin	0.10	August			100	91	100
*Carbamate*							
Bendiocarb	0.10	August			100	100	100
*Organophosphate*							
Malathion	5.0	August			100	91	100

Among the village-wise pools of female *P. argentipes* sandflies tested by PCR for *L. donovani* parasite DNA, 66.7% (95% CI = 47.8-85.5) were positive in the VL high endemic village, 62.1% (95% CI = 44.4- 79.7) in the VL moderate endemic village, 36.8% (95% CI = 15.1- 58.5) in the low endemic village, and 26.3% (95% CI = 6.5- 46.1) in the non-endemic village. Overall, 50.5% (95% CI = 40.2- 60.8) of the total 91 female *P. argentipes* sandfly pools tested were positive for parasite DNA (Table 4 and S1 Fig).

**Table 4:** Xenomonitoring results of female *P. argentipes* sandflies collected in villages with different levels of VL endemicity.

Villages	No. of pools of female *P. argentipes* tested	No. of pools positive for *L. donovani* DNA	Percentage of positive pools (95% CI)
Ishworpur Ward No. 2 (Writerkhor) (High endemic)	24	16	66.7 (47.8- 85.5)
Kabilasi Ward No. 10 (Salimpur) (Moderate endemic)	29	18	62.1 (44.4- 79.7)
Bagmati Ward No. 9 (Shankarpur) (Low endemic)	19	7	36.8 (15.1- 58.5)
Kadauna Ward No. 4 (Motipur) (Non-endemic)	19	5	26.3 (6.5- 46.1)
Total	91	46	50.5 (40.2- 60.8)

### VL case occurrence and IRS programmes history

Cumulative number of VL cases over the last ten years were, maximum 30 in Ishworpur municipality, 11 in Kabilashi municipality, 3 in Bagmati municipality and no cases in Kadauna rural municipality (S4 Table).

IRS was not conducted in any village within Sarlahi district during the years 2019, 2020, and 2021 because of re-structuring of the health system due to federalization in the country, and disruptions of public health programme due to COVID-19. However, in 2022, IRS operations were carried out in 2 out of 5 villages affected by VL, covering 2,130 households, 330 cattle sheds, and a population of 14,660. Moreover, no long-lasting insecticidal nets (LLINs) were distributed in Sarlahi district over the past decade (S5 Table). Pregnant women and VL patients received one LLIN each from government of Nepal but others used commercially available ordinary bednets. None of the bednets were more than 3–4 years old.

There was no IRS in Kadauna rural municipality for the last 10 years. In Bagmati municipality, the last IRS was conducted 2 years ago. In Kabilashi municipality, the last spraying was done 9 years ago, while in Ishworpur municipality, it was carried out 3 years ago (Table 5).

**Table 5:** Status of IRS in the selected study villages of different municipalities.

Year (Nepali fiscal year)	Ishworpur municipality	Kabilashi municipality	Bagmati municipality	Kadauna rural municipality
2014 (070/071)	Spraying conducted	Spraying conducted	No spraying	No spraying until 2022
2015 (071/072)	Spraying conducted	Spraying conducted	No spraying	No spraying until 2022
2016 (072/073)	Spraying conducted	No spraying	Spraying conducted	No spraying until 2022
2021 (077/078)	Spraying conducted	No spraying	Spraying conducted	No spraying until 2022
2022 (078/079)	No spraying	No spraying	Spraying conducted	No spraying until 2022

The district health officer and focal person of VL at the district mentioned that IRS should be continued in VL case detected villages ensuring its quality. Earlier there were high numbers of VL cases in Sarlahi district. The decrease in VL cases with few numbers in recent years can be attributed to IRS since IRS was performed two rounds per year for three consecutive years in VL case reported village. Active case detection of VL, vector control, and case diagnosis and treatment were the only measures applied for VL prevention and control in Sarlahi district. The improvement of housing and sanitation in the village might also contribute to the reduction in VL cases.

## Discussion

In this study, we compared programmatic, behavioural and entomological determinants relevant to IRS for VL control across four villages, one village per study arm selected based on differing levels of endemicity to answer the research question of when IRS can be safely discontinued. The number of reported VL cases within the Sarlahi district over the past decade, categorized by municipality revealed disparities in IRS implementation and the impact of programmatic and peoples’ behavioural factors between areas consistently affected by VL and those that have been free of VL for many years. Additionally, contrasting areas with ongoing VL transmission against those without cases for an extended period provided valuable insights into the factors influencing disease transmission and control efforts.

In this study, 57.6% of people of VL endemic villages were observed to sleep on the floor. Houses were made of branches and mud in VL high endemic villages as compared to villages with low or not endemic to VL. Mud walls with cracks and holes, and damp houses are known risk factors for VL [36]. These housing characteristics create a favorable environment for sandfly breeding and resting [37,38]. Housing improvement as well as raising awareness could complement vector control, reducing the need of IRS. Similarly, we found that bednet use in households was 49.5% in high and 56.4% in moderate endemic villages as compared to low (100%) or non- endemic (91.2%) villages. Insecticide impregnated or non-impregnated common bednets are both protective against sandfly bites and are very effective in the control of VL [39–42]. This suggests that personal protective measures including bednet use could be promoted and further assessed to replace vector control by IRS.

The outside sleeping habits particularly in the balcony during the summer season is also a risk factor for VL. There was a significant difference in sleeping habits of people in VL endemic and non-endemic villages. IRS in the inner wall of the house only is not sufficient to control sandflies. Sandflies were also collected resting above six feet and outdoor. This indicates that IRS should also be conducted in possible resting sites of sandflies around the house in addition to inner walls of the house.

The awareness of VL among community people was low. This could be the reason of risk behaviour such as low bednet use throughout the year in high endemic villages, and not keeping the house clean. Lack of community awareness in VL endemic villages has been identified as a major challenge in VL control and elimination [43].

Analysis of reported VL cases in Sarlahi district over the past decade revealed a very distinct situation on VL case occurrence. This could be due to differences in IRS application and programmatic factors across municipalities. No IRS was conducted in some villages over the past decade, and there was variations in coverage and frequency of IRS in some villages. Occupational distribution varies across endemicity levels, with differences observed in sleeping habits and potential risk of VL transmission during warm weather. One study in India revealed that the intensified control approach comprising indoor residual spraying with improved supervision; VL-specific training for accredited social health activists to reduce onset-to-diagnosis time; and increased Information Education and Communication activities in the community may have precipitated a substantial change in VL incidence [44]. Regarding IRS activities, these should be complete and coverage should be high [45,46] and the national programme should conduct monitoring and evaluation activities to ensure high quality of IRS operations to achieve sustained reduction of vector densities [47]. The analysis of programmatic IRS implementation also revealed reduction in indoor sandfly abundance contributing to the reduction of VL cases and related deaths [20].

In this study, the temperature and humidity were found favourable in all areas for the sandfly vector and infectivity of *Leishmania* parasites and this was also reported in a previous study [48]. Monthly density of female *P. argentipes,* sandflies varied across endemicity levels, with fluctuations observed throughout the year, indicating different seasonal patterns of vector abundance. The sandfly abundance was found increasing from April with a peak in November in all endemicity villages. In a previous study the seasonality of *P. argentipes* was found to be similar in India and Nepal, with two annual density peaks around May and October. Monthly *P. argentipes* density was positively associated with temperature and negatively associated with rainfall in both study sites [49]. This is similar to what was observed in a longitudinal study in Sri Lanka which revealed that increase in rainfall and relative humidity at real time, and ambient temperature and soil temperature with a 2-month lag were associated with an increase in sand fly density [50].

The results of this study demonstrated high susceptibility of *P. argentipes* to alpha-cypermethrin (0.1%), deltamethrin (0.05%), bendiocarp (0.10%), and malathion (5.0%), indicating these insecticides remain effective for controlling sandfly populations in the study area. Previous study in Nepal reported that *P. argentipes* sandflies have emerged with pyrethroid resistance and suggested to switch to alternative classes of insecticides for IRS [28]. These findings highlight the need for continuous susceptibility monitoring to detect potential resistance development, especially with the increased use of pyrethroids.

In this study, more than 50% of village wise pools of sandflies were found to be positive for *L. donovani* parasite DNA. This indicates that a high percentage of sandfly pools are infectious in high and moderate endemic villages. A xenomonitoring study conducted in India revealed that *P. argentipes* from Bihar, West Bengal, and Kerala exhibited high levels of *L. donovani* parasite DNA [51]; however, another study conducted in India reported only 0.03% infection rate in sandflies [23]. It indicates that despite the low number of human VL cases, there is a persistent transmission risk, and that the parasite continues to circulate in these areas. It might be driven by asymptomatic cases of VL or post kala-azar dermal leishmaniasis (PKDL) or the VL cases have not been captured by the passive surveillance system. The transmission chain has not been broken down and VL can resurge if IRS is stopped as reported from India [22]. Therefore, it is suggested to conduct comprehensive vector surveillance and perform effective vector control measures such as IRS and effective disease surveillance to prevent the occurrence of VL for achieving its elimination.

We conducted this study for 12 months beginning from March 2023 to February 2024. The last IRS was conducted in Bagmati municipality in 2022, while no IRS has been conducted in Kaduana municipality for the last ten years. There was no IRS in Ishworpur and Kabilashi municipality in 2022. LLINs were not distributed to these populations and people were found using ordinary bednets. IRS activities including other preventive public health programs were sub-optimal in Nepal because of the re-structuring of the health system due to federalization after 2015 and later due to the impact of COVID-19 in 2020–2021. Vector density analysis in 2023 showed that there was high density of sandflies and there was no effect of past IRS. It is obvious that IRS is effective for six months and there is a recommendation to perform IRS twice a year, first round in April-May and second round in October-November [52]. The study conducted in India revealed that combination of effective disease surveillance, case management and vector control through IRS keeps *P. argentipes* abundance and their infectivity low thus reducing VL transmission [23]. Since the effectiveness of IRS is for only a short period of time, other measures are equally important along with focused IRS using better products.

The study has some limitations. We selected four villages of one district with only one village per endemicity level of VL to perform data collection. For the sandfly abundance study, households were selected differently to place CDC light traps in VL endemic and non-endemic villages and this might have potentially biased the results. The variation in IRS application, programmatic factors and risk factors within four villages indicate that the results are limited to only similar settings. Further, we were not able to fully answer the research question on whether IRS can be safely discontinued. We did not have baseline data and data on quality of IRS conducted in the past to measure the impact of IRS. There might be many unknown factors to be explored and require statistical modeling to reach the robust conclusion.

Overall, the findings of this study contribute to a comprehensive understanding of the epidemiological, ecological and programmatic aspects in VL transmission. This provides valuable information for designing public health interventions and enhancing VL elimination efforts emphasizing targeted strategies and continued community engagement in endemic areas. Even after the elimination, there will be potential for low level transmission of the VL. Therefore, it requires effective disease surveillance through active case detection, and immediate response activities through focal IRS for vector control similar to other vector borne diseases.

In conclusion, sandflies are still susceptible to the standard insecticides. High proportion of sandflies are parasite DNA positives indicating infectiousness. There is a variation in IRS application and programmatic factors across different municipalities/villages. Some behaviours identified constitute risk factors for vector borne disease transmission. IRS still remains a cornerstone for VL elimination, specifically targeting sandflies with indoor resting habits. IRS might not be required only when the abundance of sandflies is very low and there is zero transmission of VL. If the VL prevalence is low in humans, the only known reservoir in this region, the basic reproduction number could be below one which indicates that there will be no requirement of IRS. Therefore, robust surveillance of VL and reaction based effective IRS and response should be continued until achieving and sustaining zero transmission of VL.

## Supporting information

S1 Table Characteristics of the study populations in villages with varying VL endemicity. (DOCX)

S2 Table Total number of rooms and bedrooms in households in VL endemic and non-endemic villages. (DOCX)

S3 Table Month wise mean temperature and humidity in four villages. (DOCX)

S4 Table VL cases in Sarlahi district within past 10 years. (DOCX)

S5 Table Status of vector control interventions in Sarlahi district (2012–2022). (DOCX)

S1 Fig Agarose gel electrophoresis photo of PCR products of L. donovani parasite DNA after xenomonitoring PCR (Lane 1: 100 bp marker, lane 2: positive control, lane 3, 10, 11, 12, 20: positive pool of 720 bp, lane 4, 5, 6, 7, 8, 9, 13, 14, 15, 16, 17, 18, 19: negative pool). (TIF)
