# Supplementary material for: Decision-making for indoor residual spraying in the post-elimination phase of visceral leishmaniasis in Nepal
Source: PLoS Negl Trop Dis. 2026 May 18;20(5):e0014355. doi: 10.1371/journal.pntd.0014355 (PMC13197072; doi:10.1371/journal.pntd.0014355)
Supplement: S1 Table — (DOCX) [file pntd.0014355.s001.docx]

**Supplementary file**

Supplementary table 1: Characteristics of the study populations in villages with varying VL endemicity

| Characteristics | Ishworpur  Ward No. 2 (Writerkhor)  (High endemic) | Kabilasi  Ward No. 10 (Salimpur)  (Moderate endemic) | Bagmati  Ward No. 9 (Shankarpur)  (Low endemic) | Kadauna  Ward No. 4 (Motipur)  (Non-endemic) |
| --- | --- | --- | --- | --- |
| *n* | 99 | 133 | 171 | 251 |
| Households screened (n, %) | 17 (14.0) | 54 (44.6) | 27 (22.3) | 23 (19.0) |
| *Age distribution in years (%)* |  |  |  |  |
| ≤ 10 | 27.3 | 36.8 | 17.5 | 27.9 |
| 11-20 | 24.2 | 17.3 | 22.8 | 19.1 |
| 21-45 | 31.3 | 32.3 | 40.9 | 35.5 |
| 46-60 | 12.1 | 12.8 | 12.9 | 12.7 |
| > 60 | 5.1 | 0.8 | 5.8 | 4.8 |
| *Gender (%)* |  |  |  |  |
| Male | 54.5 | 50.4 | 52.6 | 51.4 |
| Female | 45.5 | 49.6 | 47.4 | 48.6 |
| *Occupation (%)* |  |  |  |  |
| Farmer | 1.0 | 11.3 | 9.3 | 0.8 |
| Business owner | 0 | 0 | 2.9 | 1.2 |
| Migrant worker | 0 | 0.8 | 5.8 | 1.6 |
| Govt. employee | 0 | 0 | 1.8 | 0 |
| Private employee | 0 | 0 | 1.8 | 0 |
| Skilled worker | 1.0 | 0.8 | 8.2 | 3.6 |
| Unskilled worker | 30.3 | 27.8 | 0 | 22.7 |
| Housewife | 23.2 | 24.0 | 25.1 | 26.3 |
| Student | 12.1 | 6.0 | 31.6 | 18.3 |
| Children | 11.1 | 28.5 | 8.8 | 14.3 |
| Others | 21.2 | 0.8 | 4.7 | 11.2 |
| *Place of sleeping* |  |  |  |  |
| Bed | 37.4 | 27.1 | 100 | 85.7 |
| Floor | 54.5 | 55.6 | 0 | 11.5 |
| Both | 8.1 | 17.3 | 0 | 2.8 |
| *Slept under bednet last night (%)* |  |  |  |  |
| Yes | 49.5 | 56.4 | 100 | 91.2 |
| No | 50.5 | 43.6 | 0 | 8.8 |
