# Supplementary material for: Decision-making for indoor residual spraying in the post-elimination phase of visceral leishmaniasis in Nepal
Source: PLoS Negl Trop Dis. 2026 May 18;20(5):e0014355. doi: 10.1371/journal.pntd.0014355 (PMC13197072; doi:10.1371/journal.pntd.0014355)
Supplement: S2 Table — (DOCX) [file pntd.0014355.s002.docx]

Supplementary table 2: Total number of rooms and bedrooms in households in VL endemic and non-endemic villages

| Characteristics (*n*, %) | Ishworpur Ward No. 2 (Writerkhor)  (High endemic) | Kabilasi Ward No. 10 (Salimpur)  (Moderate endemic) | Bagmati  Ward No. 9 (Shankarpur)  (Low endemic) | Kadauna  Ward No. 4 (Motipur)  (Non-endemic) | Endemic  (n=98) | Non-endemic (n=23) | p-value |
| --- | --- | --- | --- | --- | --- | --- | --- |
| *No. of rooms in the households (HHs)* |  |  |  |  |  |  |  |
| 1-2 rooms | 12 (70.6) | 28 (51.9) | 1 (3.7) | 14 (60.9) | 41 (41.8) | 14 (60.9) | 0.05* |
| 3-5 rooms | 3 (17.6) | 23 (42.6) | 9 (33.3) | 8 (34.8) | 35 (35.7) | 8 (34.8) |  |
| ≥6 rooms | 2 (11.8) | 3 (5.6) | 17 (63.0) | 1 (4.3) | 22 (22.4) | 1 (4.3) |  |
| *Median no. of rooms per HH* | 2 (1-7) | 2 (1-6) | 6 (2-13) | 2 (1-7) | 3 (1-13) | 2 (1-7) | 0.04^$^ |
| *No. of bedrooms in the HHs* |  |  |  |  |  |  |  |
| 1 bedroom | 8 (47.1) | 23 (42.6) | 2 (7.4) | 11 (47.8) | 33 (33.7) | 11 (47.8) | 0.42* |
| 2-3 bedrooms | 8 (47.1) | 28 (51.8) | 15 (55.6) | 10 (43.5) | 51 (52.0) | 10 (43.5) |  |
| ≥4 bedrooms | 1 (5.8) | 3 (5.6) | 10 (37.0) | 2 (8.7) | 14 (14.3) | 2 (8.7) |  |
| *Median no. of bedrooms per HH* | 2 (1-6) | 2 (1-4) | 3 (1-8) | 2 (1-4) | 2 (1-8) | 2 (1-4) | 0.17^$^ |
| *Type of kitchen* |  |  |  |  |  |  |  |
| Within a bedroom | 13 (76.5) | 27 (50.0) | 1 (3.7) | 12 (52.2) | 41 (41.8) | 12 (52.2) | 0.53* |
| Separate room | 3 (17.6) | 22 (40.7) | 26 (96.3) | 9 (39.1) | 51 (52.0) | 9 (39.1) |  |
| Open space | 1 (5.9) | 5 (9.3) | 0 (0.0) | 2 (8.7) | 6 (6.1) | 2 (8.7) |  |
| *Cattle shed at the house* |  |  |  |  |  |  |  |
| Yes | 3 (17.6) | 25 (46.3) | 25 (92.6) | 12 (52.2) | 53 (54.1) | 12 (52.2) | 0.87^ |
| No | 14 (82.4) | 29 (53.7) | 2 (7.4) | 11 (47.8) | 45 (45.9) | 11 (47.8) |  |

* Likelihood ratio chi-square test

$ Mann-Whitney U test

^ Fisher’s exact test
