# Supplementary material for: Decision-making for indoor residual spraying in the post-elimination phase of visceral leishmaniasis in Nepal
Source: PLoS Negl Trop Dis. 2026 May 18;20(5):e0014355. doi: 10.1371/journal.pntd.0014355 (PMC13197072; doi:10.1371/journal.pntd.0014355)
Supplement: S3 Table — (DOCX) [file pntd.0014355.s003.docx]

Supplementary table 3: Month wise mean temperature and humidity in four villages

| Months | Ishworpur Ward No. 2 (Writerkhor)  (High endemic) | | Kabilasi Ward No. 10 (Salimpur)  (Moderate endemic) | | Bagmati Ward No. 9 (Shankarpur)  (Low endemic) | | Kadauna Ward No. 4 (Motipur)  (Non-endemic) | |
| --- | --- | --- | --- | --- | --- | --- | --- | --- |
|  | Temp. (^o^C) | Humidity | Temp. (^o^C) | Humidity | Temp. (^o^C) | Humidity | Temp. (^o^C) | Humidity |
| January | Min.=9.0  Max.=24.0 | Min.=72.0  Max.=84.0 | Min.=13.0  Max.=28.0 | Min.=80.0  Max.=82.0 | Min.=15.0  Max.=18.3 | Min.=62.0  Max.=71.0 | Min.=15.0  Max.=28.0 | Min.=47.0  Max.=63.0 |
| February | Min.=21.2  Max.=27.0 | Min.=59.0  Max.=85.0 | Min.=22.0  Max.=27.9 | Min.=59.0  Max.=85.0 | Min.=21.0  Max.=27.9 | Min.=59.0  Max.=85.0 | Min.=21.9  Max.=27.9 | Min.=59.0  Max.=85.0 |
| March | Min.=14.3  Max.=25.1 | Min.=34.0  Max.=74.0 | Min.=13.5  Max.=27.7 | Min.=21.0  Max.=85.0 | Min.=13.5  Max.=27.6 | Min.=23.0  Max.=88.0 | Min.=13.5  Max.=26.6 | Min.=37.0  Max.=84.0 |
| April | Min.=26.5  Max.=27.1 | Min.=68.0  Max.=72.0 | Min.=28.0  Max.=30.0 | Min.=60.0  Max.=65.0 | Min.=27.6  Max.=28.1 | Min.=66.0  Max.=71.0 | Min.=27.1  Max.=33.0 | Min.=36.0  Max.=68.0 |
| May | Min.=31.6  Max.=32.0 | Min.=47.0  Max.=49.0 | Min.=30.8  Max.=31.0 | Min.=61.0  Max.=66.0 | Min.=32.2  Max.=32.6 | Min.=55.0  Max.=56.0 | Min.=30.5  Max.=32.8 | Min.=62.0  Max.=83.0 |
| June | Min.=26.0  Max.=36.0 | Min.=78.0  Max.=92.0 | Min.=26.0  Max.=33.0 | Min.=51.0  Max.=72.0 | Min.=26.0  Max.=32.0 | Min.=89.0  Max.=92.0 | Min.=25.0  Max.=29.0 | Min.=94.0  Max.=96.0 |
| July | Min.=28.0  Max.=30.0 | Min.=84.0  Max.=87.0 | Min.=29.0  Max.=31.4 | Min.=74.0  Max.=77.0 | Min.=28.0  Max.=30.0 | Min.=86.0  Max.=90.0 | Min.=27.0  Max.=29.0 | Min.=86.0  Max.=90.0 |
| August | Min.=27.0  Max.=34.0 | Min.=60.0  Max.=77.0 | Min.=27.0  Max.=35.0 | Min.=60.0  Max.=67.0 | Min.=27.0  Max.=34.0 | Min.=65.0  Max.=73.0 | Min.=28.0  Max.=36.0 | Min.=63.0  Max.=68.0 |
| September | Min.=27.3  Max.=28.4 | Min.=70.0  Max.=76.0 | Min.=27.2  Max.=30.1 | Min.=77.0  Max.=99.0 | Min.=27.8  Max.=28.2 | Min.=75.0  Max.=80.0 | Min.=27.4  Max.=36.1 | Min.=59.0  Max.=92.0 |
| October | Min.=19.4  Max.=23.5 | Min.=62.0  Max.=74.0 | Min.=26.0  Max.=26.4 | Min.=63.0  Max.=70.0 | Min.=22.6  Max.=23.2 | Min.=71.0  Max.=72.0 | Min.=24.4  Max.=29.9 | Min.=59.0  Max.=84.0 |
| November | Min.=13.0  Max.=25.0 | Min.=75.0  Max.=81.0 | Min.=17.0  Max.=27.0 | Min.=44.0  Max.=66.0 | Min.=11.0  Max.=24.0 | Min.=76.0  Max.=82.0 | Min.=16.0  Max.=22.0 | Min.=64.0  Max.=76.0 |
| December | Min.=16.9  Max.=17.1 | Min.=80.0  Max.=80.0 | Min.=15.9  Max.=22.4 | Min.=47.0  Max.=54.0 | Min.=8.9  Max.=11.7 | Min.=59.0  Max.=72.0 | Min.=15.3  Max.=18.3 | Min.=67.0  Max.=70.0 |
