# Supplementary material for: Decision-making for indoor residual spraying in the post-elimination phase of visceral leishmaniasis in Nepal
Source: PLoS Negl Trop Dis. 2026 May 18;20(5):e0014355. doi: 10.1371/journal.pntd.0014355 (PMC13197072; doi:10.1371/journal.pntd.0014355)
Supplement: S4 Table — (DOCX) [file pntd.0014355.s004.docx]

Supplementary table 4: VL cases in Sarlahi district within past 10 years

| S.N. | Year | Ishworpur municipality | Kabilashi municipality | Bagmati municipality | Kadauna  rural municipality |
| --- | --- | --- | --- | --- | --- |
| 1 | 2069/2070 | 1 | - | - | - |
| 2 | 2070/2071 | - | 4 | - | - |
| 3 | 2071/2072 | - | 7 | - | - |
| 4 | 2072/2073 | 3 | - | - | - |
| 5 | 2073/2074 | - | - | - | - |
| 6 | 2074/2075 | 17 | - | - | - |
| 7 | 2075/2076 | 5 | - | 1 | - |
| 8 | 2076/2077 | 4 | - | - | - |
| 9 | 2077/2078 | - | - | 1 | - |
| 10 | 2078/2079 | - | - | 1 | - |
|  | Remarks | High endemic | Moderate endemic | Low endemic | Non-endemic |
