# Supplementary material for: Decision-making for indoor residual spraying in the post-elimination phase of visceral leishmaniasis in Nepal
Source: PLoS Negl Trop Dis. 2026 May 18;20(5):e0014355. doi: 10.1371/journal.pntd.0014355 (PMC13197072; doi:10.1371/journal.pntd.0014355)
Supplement: S5 Table — (DOCX) [file pntd.0014355.s005.docx]

Supplementary table 5: Status of vector control interventions in Sarlahi district (2012-2022)

| Year | No. of VL  affected  villages | No. of VL  non-affected  villages | IRS  performed | No. of  IRS  villages | No. of households  covered  by IRS | Population (No. of inhabitants)  covered  by IRS | No. of cattle shed  covered  by IRS |
| --- | --- | --- | --- | --- | --- | --- | --- |
| 2012 | 16 | 85 | Yes | 6 | 7440 | 43160 | 1410 |
| 2013 | 12 | 89 | Yes | 3 | 5784 | 43090 | 1100 |
| 2014 | 15 | 86 | Yes | 5 | 9115 | 61836 | 1636 |
| 2015 | 10 | 91 | Yes | 5 | 6060 | 36970 | 1280 |
| 2016 | 15 | 86 | Yes | 10 | 12138 | 74466 | 2572 |
| 2017 | 14 | 87 | Yes | 4 | 5045 | 27310 | 944 |
| 2018 | 12 | 89 | Yes | 5 | 4823 | 28603 | 922 |
| 2019 | 0 | 0 | No | 0 | 0 | 0 | 0 |
| 2020 | 0 | 0 | No | 0 | 0 | 0 | 0 |
| 2021 | 0 | 0 | No | 0 | 0 | 0 | 0 |
| 2022 | 5 | 15 | Yes | 2 | 2130 | 14660 | 330 |
